# Spectral interpretation of late-stage mare basalt mineralogy unveiled by Chang’E-5 samples

**DOI:** 10.1038/s41467-022-33670-6

**Published:** 2022-10-10

**Authors:** Dawei Liu, Xing Wang, Jianjun Liu, Bin Liu, Xin Ren, Yuan Chen, Zhaopeng Chen, Hongbo Zhang, Guangliang Zhang, Qin Zhou, Zhoubin Zhang, Qiang Fu, Chunlai Li

**Affiliations:** 1 Key Laboratory of Lunar and Deep Space Exploration, National Astronomical Observatories, Chinese Academy of Sciences, Beijing, 100101 China; 2 School of Astronomy and Space Science, University of Chinese Academy of Sciences, Beijing, 100049 China

**Keywords:** Planetary science, Planetary science

## Abstract

The western maria of lunar near-side are widely covered with late-stage mare basalts. Due to the lack of returned samples, the mineralogy of the late-stage basalts was previously speculated as having high abundance of olivine based on remote sensing observation. However, here we show that Chang’E-5 (CE-5) lunar soil samples, the ground truth from past unsampled lunar late-stage mare region, give a different interpretation. Our laboratory spectroscopic and X-ray diffraction (XRD) analyses of the CE-5 soil samples demonstrate that their special spectral signatures are representative of iron-rich high-Ca pyroxene rather than olivine. Considering the spectral and compositional similarities between CE-5 soil samples and lunar late-stage basalts, the mineralogy and petrology of CE-5 samples may be able to be generalized to entire lunar late-stage basalts. Our study would provide a constraint on the thermal evolution of the Moon, especially the young lunar volcanism.

## Abstract (web summary)

Laboratory spectroscopic and X-ray diffraction analyses on the Chang’E-5 lunar soil samples have demonstrated that the spectral features of lunar late-stage mare basalts are representative of iron-rich high-Ca pyroxene rather than olivine.

## Introduction

Lunar mare basalts, as an important product of lunar volcanism, cover about 17% of the total lunar surface area^1^. Analysis of basalt samples returned by the Apollo and Luna missions suggests that lunar mare volcanism was active between ~4.3 Ga and 3.1 Ga^2,3^. Crater size-frequency distribution (CSFD) measurements based on remote sensing data indicate that although most lunar volcanism occurred at 3.6–3.8 Ga during the Late Imbrian (Im) period, lunar volcanism continued to at least 1.2 Ga^4–6^. The basalts released by these late-stage volcanisms are mainly distributed in Oceanus Procellarum (OP) and Mare Imbrium^4,5^. They are distinguishable dark volcanic flows with medium to high titanium contents and also relative high iron contents^7–12^(Supplementary Fig. 1a–c). Understanding their petrogenesis is of significant importance for understanding the late-stage thermal evolution of the Moon. However, due to the lack of returned samples, the composition of late-stage mare basalts can be only inferred from spectral analysis of remote sensing data^13–15^. Lunar late-stage basalts have been found to have distinctive spectral features, either through Earth-based telescope^16,17^ data or through multispectral (e.g., Clementine)^13,15^ and hyperspectral (e.g., Moon Mineralogy Mapper (M^3^))^14,18–21^ orbital data. Their 1 μm absorption is broad and asymmetric, while their 2 μm absorption is much weaker. Moreover, the band centers of the late-stage basalts shift toward the longer wavelengths. Such spectral signatures have been speculated as having a high abundance of olivine, even indicating the abundance ratio of olivine to pyroxene could exceed 1^14,16–18^. Also, due to their distinctive spectral characteristics, the late-stage basalts appear in red hue on the integrated band depth (IBD) map derived from M^3^ data, while nearby older mare regions are in green-yellow hue (Supplementary Fig. 1d). According to the inferred mineral features of enriching olivine, Staid et al.^14^ proposed that the Fe-rich olivine in the late-stage mare basalts may originate through the crystallization of an evolved residual melt rather than through the assimilation of more primitive (Mg-rich) olivine-rich sources. Furthermore, Chang’E-3 (CE-3) landed at north Mare Imbrium (340.49°E, 44.12°N) a region likely to be covered by late-stage mare basalts^4,22,23^. This landing site is also thought to have high olivine abundance^24^. Based on CE-3 in-situ measurements from the onboard Visible and Near-Infrared Imaging Spectrometer and Active Particle-Induced X-ray Spectrometer, Ling et al.^24^ proposed that the source of olivine-rich young mare deposits may be formed during late-stage lunar magma ocean differentiation, when dense ferropyroxene-ilmenite cumulates sank and mixed with deeper, ferroan olivine and orthopyroxene in a hybridized mantle source^25^. However, these speculations about the petrogenesis of the late-stage mare basalts are based on the fact that they have an elevated olivine abundance. Since the relevant ground truth was absent, it remains unconfirmed whether late-stage mare basalts are indeed rich in olivine as postulated by remote sensing observations.

**Fig. 1:**
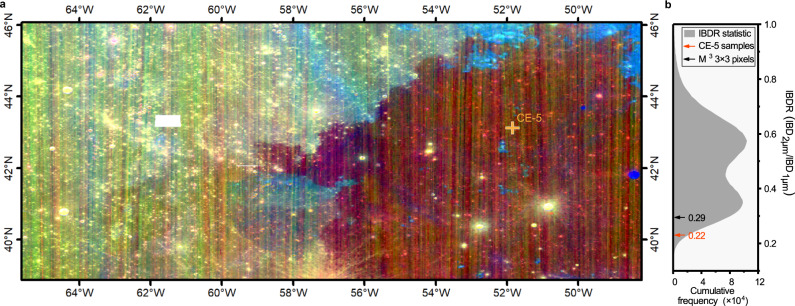
Moon Mineralogy Mapper (M^3^) integrated band depth (IBD) map of the CE-5 landing area and its integrated band depth ratio (IBDR) statistic. **a** M^3^ IBD map of the CE-5 landing area (R: IBD_1μm_, G: IBD_2μm_, B: 1500 nm reflectance). Eratosthenian (Em) late-stage mare (P58 unit) shows a red hue while Imbrian (Im) old mare to the west of the CE-5 landing site shows a green-yellow hue. The solid cross indicates the CE-5 landing site. **b** The IBDR statistic of P58 unit and adjacent older mare basalts to the west. The IBDR statistic shows a bimodal distribution. IBDRs of P58 unit are mostly concentrated in the lower peak, while the IBDRs of older basalts to the west are mostly concentrated in the upper peak. The IBDR of CE-5 samples (orange arrow) is small (0.22) within the range of P58 unit, and is consistent with the IBDR value (0.29, black arrow) of the average spectrum derived from 3 × 3 M^3^ pixels area around CE-5 landing site. The small white rectangle in the map is not covered by M^3^ data.

On December 1, 2020, Chinese CE-5 spacecraft successfully landed on the mare plain in the northeastern part of OP. The landing site (51.916°W, 43.058°N) is located in an Eratosthenian (Em) mare unit (named as P58 in ref. 5) which is one of the youngest basaltic regions on the lunar surface^5,22,26–29^ (Fig. 1a). The two-billion-year-old sample^30,31^ returned by CE-5 mission provides an opportunity to reconnect with the lunar late-stage mare volcanism.

Here, we show that the spectral features of CE-5 returned lunar soil samples are representative of iron-rich high-Ca pyroxene rather than olivine based on laboratory measurements (see Methods section for detailed sample preparation, spectroscopic, and XRD measurements). Spectral and compositional similarity between CE-5 samples and late-stage mare basalt regions indicates their similar mineralogy and petrology.

## Results and discussion

### Laboratory spectroscopic analysis of Chang’E-5 soil samples

**Fig. 2:**
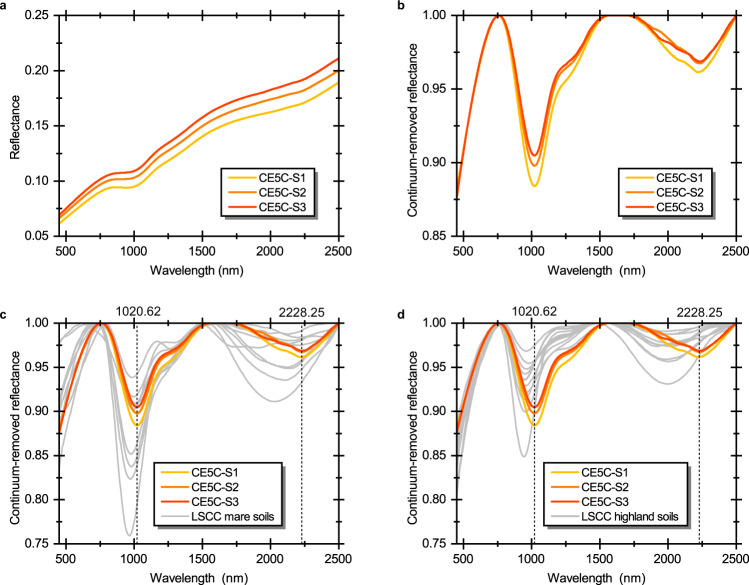
Spectral shape comparison between CE-5 and Lunar Soil Characterization Consortium (LSCC) soil samples. **a** Laboratory measured spectra of three CE-5 lunar soil samples. **b** Continuum-removed spectra of CE-5 samples. **c** Spectra comparison between CE-5 and LSCC mare soil samples. **d** Spectra comparison between CE-5 and LSCC highland soil samples. Dashed lines and numbers in **c**, **d** represent the average 1 μm and 2 μm band centers of three CE-5 soil samples.

**Fig. 3:**
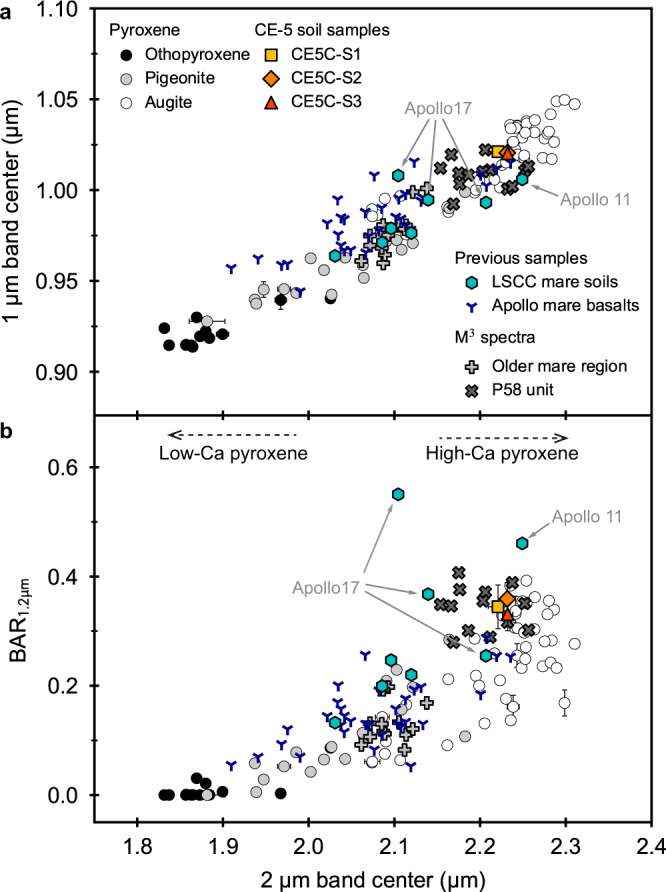
Spectral parameters comparison of the CE-5 samples with pure pyroxenes, previous mare soil and basalt samples, and Moon Mineralogy Mapper (M^3^) orbital spectra. **a** 1 μm and 2 μm band centers. **b** 1.2 μm band area ratio (BAR_1.2μm_) and 2 μm band center. How to derive these spectral parameters (1 μm and 2 μm band centers, BAR_1.2μm_) can be found in the Methods section. The selection of these pure pyroxenes is described in Supplementary Note 1. The error bars of some pure pyroxene points indicate the standard deviation of the samples with different grain sizes, and the points without error bars are derived from a single measurement. The error bars of CE-5 soil samples are the standard deviation of the derived spectral parameters. The spectral parameters of M^3^ data correspond to the spectra shown in Fig. 6. Note that the spectral parameters of Lunar Soil Characterization Consortium (LSCC) 71061 sample are not shown in the figure, because it shows uncommon spectral characteristics due to the high abundance of volcanic glass. Solid gray arrows indicate the LSCC samples from Apollo 11 and Apollo 17. Dashed arrows represent direction of dominant pyroxene composition.

We performed spectroscopic measurement on three CE-5 lunar soil samples in laboratory, which are CE5C0800YJFM001-1, CE5C0100YJFM002-1 and CE5C0100YJFM002-2 (hereinafter referred as CE5C-S1, CE5C-S2, and CE5C-S3, respectively). As shown in Fig. 2a, the overall spectral shape of three samples is essentially consistent, indicating that the mineral abundances of three samples should be similar. The corresponding continuum removed spectra demonstrate their absorption features more clearly (Fig. 2b). Their 1 μm absorptions are wide and asymmetric due to the presence of prominent absorptions around 1.2 μm, and they all have weaker 2 μm absorptions relative to 1 μm absorptions. Such spectral characteristics are in agreement with the spectral signature of late-stage basalts as observed from remote sensing data^14,16–18^. Figure 3a and Table 1 show that the 1 μm and 2 μm band centers, the basic spectral parameters to identify the pyroxene-bearing materials, of CE-5 samples occur at longer wavelengths, suggesting that the bulk composition of pyroxene composing CE-5 soils should be augitic^32^. Since the area of 2 μm band is much smaller than the 1 μm band, all three CE-5 samples have small IBD_2μm_/IBD_1μm_ (IBDR) values (Table 1), which falls within the IBDR range of late-stage mare basalts covering the landing site (Fig. 1b).

**Table 1:** Spectral parameters of the three CE-5 soil samples

Spectral parameters	CE5C-S1 (STD)	CE5C-S2 (STD)	CE5C-S3 (STD)	Mean
1 μm band center (nm)	1021.15 (1.34)	1020.50 (1.90)	1020.21 (2.39)	1020.62
2 μm band center (nm)	2220.89 (11.55)	2231.73 (5.50)	2232.13 (6.06)	2228.25
IBD_1μm_	1.74 (0.01)	1.49 (0.01)	1.37 (0.01)	1.53
IBD_2μm_	0.40 (0.11)	0.31 (0.06)	0.32 (0.07)	0.34
IBDR (IBD_2μm_/IBD_1μm_)	0.23 (0.06)	0.21 (0.04)	0.23 (0.05)	0.22
1.2 μm band area ratio (BAR_1.2μm_)	0.34 (0.04)	0.36 (0.03)	0.33 (0.03)	0.34

### X-ray diffraction analysis of Chang’E-5 soil samples

**Fig. 4:**
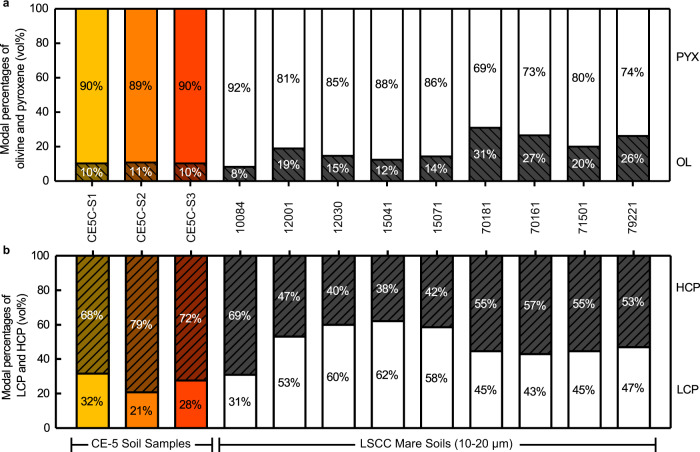
Comparison of mineral abundance ratio between CE-5 and Lunar Soil Characterization Consortium (LSCC) mare soil samples. **a** Modal percentages (vol%) of olivine (OL) and pyroxene (PYX). **b** Modal percentages (vol%) of low-Ca clinopyroxene (LCP) and high-Ca clinopyroxene (HCP). LSCC data are from ref. 44. For comparison, the sum of OL and PYX, and LCP and HCP are normalized to 100%, respectively. Given that 10–20 μm size fraction could be representative of bulk soil properties^42,43,45^, only 10–20 μm LSCC soil data are used to compare with CE-5 soil samples. The five-digit number (e.g., 10084) corresponds to the Apollo sample number.

XRD measurement was performed to obtain the phase types and their modal abundances of three CE-5 lunar soil samples. The mineral assemblage of three samples is composed of augite (high-Ca clinopyroxene, HCP), pigeonite (low-Ca clinopyroxene, LCP), plagioclase, forsterite, fayalite, ilmenite, quartz, apatite, and glassy materials^33^ (Supplementary Table 1 and 2). Pyroxene and olivine are of interest in this study because they are ferrous silicate minerals yielding the strong spectral absorption characteristics of lunar soils. The XRD result reveals that CE-5 samples are dominated by pyroxene rather than olivine, and the olivine/pyroxene (OL/PYX) ratio of CE-5 samples is quite comparable to that of Apollo mare soil samples (Fig. 4a), much <1. This is inconsistent with the past speculations of the late-stage basalt mineralogy based on remote sensing data, which were interpreted as olivine-rich or even having the olivine/pyroxene ratio over 1^14,16–18^. In terms of the pyroxene composition in CE-5 samples, HCP is more abundant than LCP. The HCP/LCP ratio is relatively high, akin to those measured in Apollo 11 high-Ti soils (Fig. 4b).

### Iron-rich high-Ca clinopyroxene dominating the spectral characteristics

**Fig. 5:**
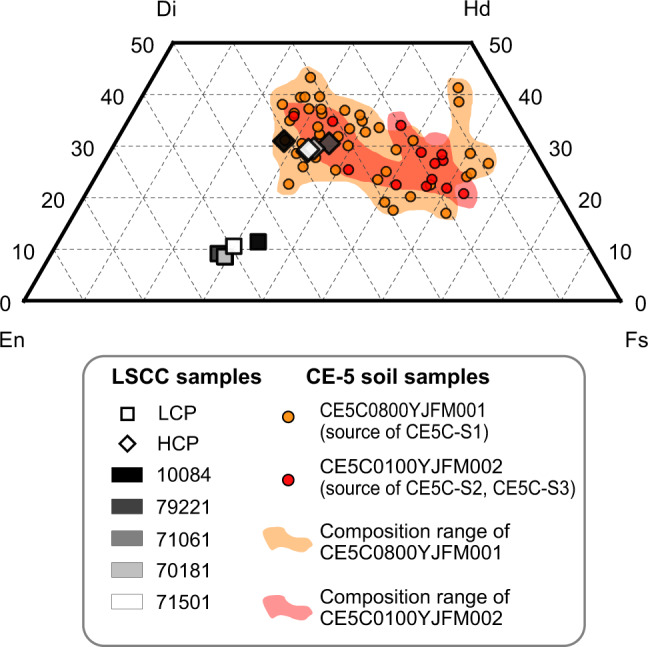
Pyroxene quadrilateral showing pyroxene compositions for CE-5 soil samples and Lunar Soil Characterization Consortium (LSCC) high-Ti mare soil samples. LSCC samples are shown in white to black color, and different geometric shapes represents different pyroxene compositions. Note that the pyroxene is divided into four types by LSCC, including orthopyroxene, pigeonite, Mg-Clinopyroxene and Fe-Clinopyroxene^44^. To simplify, we regard the weighted average composition of orthopyroxene and pigeonite as the composition of low-Ca clinopyroxene (LCP), and the weighted average composition of Mg-Clinopyroxene and Fe-Clinopyroxene as the composition of high-Ca clinopyroxene (HCP). For CE-5 samples, though we do not obtain the specific pyroxene compositions of the three spectroscopic analyzed samples, the pyroxene compositions of the two source samples were measured and could be considered as representative of the approximate compositions of these three samples. The corresponding data are from ref. 33. The pyroxene compositions of low-Ti mare soil samples are not compared because they were not provided by LSCC. The five-digit number (e.g., 10084) corresponds to the Apollo sample number. Di, Hd, En, Fs represent diopside, hedenbergite, enstatite, and ferrosilite, respectively.

Preliminary works have identified that the CE-5 returned soil sample is basically comprised of a type of lunar basalt that have never been sampled before^31,33,34^. In comparison with the mare samples collected from previous missions, the bulk composition of pyroxene in CE-5 samples (both soils and clasts) is relatively iron and calcium-rich based on electron microprobe analysis (Fig. 5 and Supplementary Fig. 2). The high HCP/LCP ratio of CE-5 samples is also revealed by the XRD measurements. The absorption features of a lunar soil spectrum are usually dominated by its mafic minerals (e.g., pyroxene and olivine) due to their strong absorption properties, while plagioclase and ilmenite have almost no strong effect on the spectral shape if their abundances are not high (e.g., plagioclase <85%^35^, ilmenite <15%^36^). The distinctive pyroxene composition and abundance in CE-5 samples will lead to a special spectral characteristic. As aforementioned, the spectra of CE-5 samples exhibit strong, broad and asymmetric 1 μm absorption (due to the prominent absorptions around 1.2 μm) and relatively weak 2 μm absorption. The 1 μm and 2 μm band centers of CE-5 samples also occur at longer wavelengths.

For pyroxene, the presence of Fe^2+^ in the crystal structure produces the near-infrared absorption bands, and the entire spectral shape varies with the populations of Fe^2+^ in the M1 and M2 octahedral cation sites^37^. The spectrum of a pure pyroxene can be deconvolved into three diagnostic absorption bands, among which the weak 1.2 μm absorption band and the 2 μm absorption band are related to the occupancy of Fe^2+^ in the M1 and M2 sites, respectively, while the 1 μm absorption band is caused by the superposition of two absorptions from both M1 and M2 sites^37–39^. In terms of the pyroxene with relatively enriched iron and calcium composition that is mostly found in CE-5 samples, larger Ca^2+^ cations prefer to occupy the M2 site and substitute for smaller Fe^2+^ cations because the M2 site is more distorted and larger than the M1 site. As Ca^2+^ is not a transition metal cation and cannot cause diagnostic absorption feature in near-infrared wavelengths, the decreasing amount of Fe^2+^ cations in M2 site leads to a weakening of 2 μm absorption band which only arises from M2 site (an example is shown in Supplementary Fig. 3). The substituted Fe^2+^ cations are forced to occupy the M1 site, strengthening the 1.2 μm absorption band which only arises from M1 site. The relatively iron- and calcium-rich pyroxene composition also shifts the 1 μm and 2 μm band centers toward longer wavelengths. Therefore, the special spectral characteristics of CE-5 samples should be attributed to that they are dominated by the HCP with such spectral signatures.

However, the spectra of late-stage basalts with spectral features similar to those of CE-5 samples have been interpreted as enriched in olivine in the past, while the XRD analysis of CE-5 samples shows the olivine abundance is not significant. What causes the misidentification of an HCP-dominated spectrum as an olivine-dominated spectrum? The spectrum of a pure olivine has a diagnostic absorption near 1.3 μm superposing with two other absorptions near 0.9 and 1.1 μm, but the 2 μm absorption band is absent^37,40^. In the case of a mixture bearing both olivine and pyroxene, if the olivine abundance is large enough, the existence of olivine can broaden the 1 μm absorption band, strengthen the 1.2 μm absorption band, and weaken the 2 μm absorption band. The resulting spectral shape is indeed easily confused with an HCP-dominated spectrum, such as the example given in Supplementary Fig. 4. Besides, the limitation of the returned samples, especially the lack of young-aged mare basalts, may also contribute to the spectral misinterpretation.

### Recognizing high-Ca clinopyroxene dominant spectra on spectral parameters

We attempt to further demonstrate that the spectral characteristics presented by CE-5 samples indicate HCP-dominated instead of olivine-rich from the perspective of spectral parameters. The commonly used relationship between 1 μm and 2 μm band centers is a valid tool to access the bulk pyroxene composition (Fig. 3a). However, it is hard to determine whether a mixture spectrum suggests the enrichment of olivine if based only on the relationship of band centers, even if the pyroxene/olivine ratio is close to 50/50 (see Supplementary Note 1 and 2). Therefore, a special spectral parameter is employed in this work, which we call as 1.2 μm band area ratio (BAR_1.2μm_, the definition is in the Methods section). BAR_1.2μm_ is associated with the 1.2 μm and 2 μm absorption bands, which are the key features causing confusion in previous studies. This spectral parameter was once used to evaluate the cooling history of pyroxenes and pyroxene-dominated rocks^41^ (known as M1 Area Ratio in ref. 41), while we apply it to the more complex olivine-bearing mixtures in this study. As described in detail in Supplementary Note 2, BAR_1.2μm_ as a function of 2 μm band center can help to distinguish whether the olivine abundance is significant in a mixture containing both olivine and pyroxene. The value of BAR_1.2μm_ will exceed 0.5 if the olivine abundance could be >50% in a mixture of olivine and HCP (Supplementary Note 2). CE-5 samples (Fig. 3b and Table 1) are at the HCP end, basically following the relationship derived from pure pyroxenes, with their BAR_1.2μm_ values not >0.5. This supports that the spectra of CE-5 samples should be dominated by HCP rather than olivine.

### Spectral comparison with samples from previous mission

LSCC has acquired typical spectra of the samples returned from each Apollo mission, and the corresponding mineral abundances and compositions are also available. Figure 2c, d present the spectral shape comparison between our three CE-5 soil samples and LSCC soil samples. Note that our spectroscopic measurement of CE-5 soil samples was performed on the bulk soil possibly with an average grain size of ~50 μm^33^, but LSCC samples were sieved into different grain size ranges (<45 μm, 20–45 μm, 10–20 μm and <10 μm). It has been demonstrated that the smaller size fractions dominate the optical properties of the bulk soil^42^. Among these size fractions of LSCC, both <45 μm and 10–20 μm fractions are considered to represent the bulk soil^42,43^. The fractions with grain size >25 μm (such as the 20–45 μm size fractions) have stronger absorption bands than the bulk soil^42^, and the optical property of the <10 μm size fractions was found to be characterized by a distinct flattening toward longer wavelengths and different from bulk soil^42,44,45^. Besides, the mineral data for <45 μm size fraction were not obtained by LSCC. In this case, we finally chose the LSCC samples in the range of 10–20 μm for comparison. Intuitively, CE-5 samples have long-wavelength band centers among these Apollo samples. This is also determined in our quantitative calculations of band centers (Fig. 3a). Apollo 11 high-Ti mare soil displays the most similar spectral parameters to CE-5 samples. The olivine abundance in both of them is not remarkable, and their OL/PYX and LCP/HCP ratios are very comparable (Fig. 4). The bulk pyroxene composition of Apollo 11 soil may be less iron-rich but is still close to those of CE-5 soil samples (Fig. 5). The band centers of Apollo 17 high-Ti mare soils also occur at relatively long-wavelength, but are shorter than CE-5 and Apollo 11 samples and longer than other low-Ti mare samples. Although Apollo 17 mare soils contain similar pyroxene composition as Apollo 11 mare soil (Fig. 5), they do not have such high HCP proportions (Fig. 4b). In addition, probably resulting from the relatively high olivine abundance (OL/PYX is nearly 30/70 shown in Fig. 4a), the BAR_1.2μm_ values of Apollo 17 mare soils tend to be large.

We also compared the spectral properties of CE-5 soil samples with some lunar mare basalts (Fig. 3). In general, the spectral characteristics of CE-5 samples are akin to some high-Ti basalts (e.g., Apollo 11 high-Ti basalt and Apollo 12 ilmenite basalt). These basalts basically present some evolved magmatic products^46^, and laboratory analysis of the CE-5 samples gave a similar interpretation^47^, though the TiO_2_ content in CE-5 samples do not reach that high.

### Spectral comparison with the P58 unit where Chang’E-5 landed

**Fig. 6:**
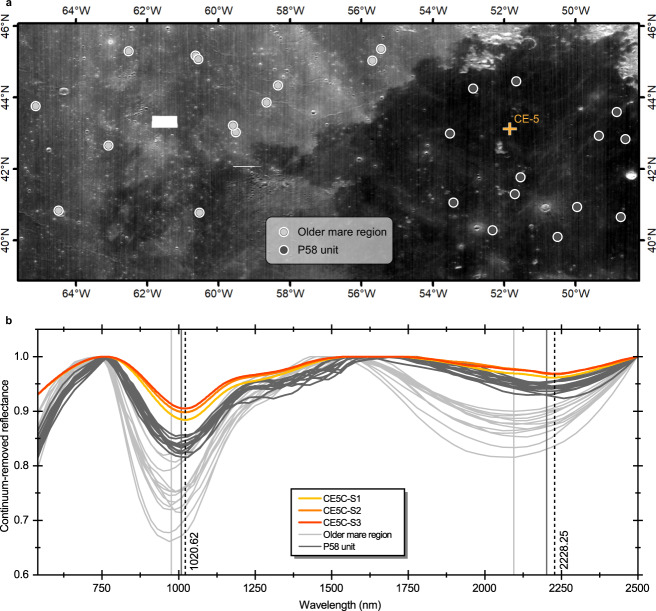
Spectral comparison of the CE-5 soil samples with P58 unit and adjacent older mare region. **a** Location of the fresh craters selected for spectral extraction. The base map is Moon Mineralogy Mapper (M^3^) 1508 nm reflectance map. **b** Continuum-removed spectra of CE-5 samples and spectra from P58 unit and adjacent older mare region. Vertical dashed lines indicate the mean band centers of CE-5 samples, and vertical gray solid lines refer to mean band centers of P58 unit and older mare region. The mean 1 μm and 2 μm band centers of the spectra in old mare region are 976.37 nm and 2095.06 nm, respectively, while the mean 1 μm and 2 μm band centers of the spectra in P58 unit are 1008.80 nm and 2202.04 nm, respectively. Numbers close to dashed line represent the average 1 μm and 2 μm band centers of three CE-5 soil samples. The small white rectangle in the map is not covered by M^3^ data.

The sampling site of the CE-5 mission is located in the area covered by Eratosthenian-aged late-stage mare basalts, which is named as P58 unit in ref. 5. Since the collected CE-5 samples are dominated by iron-rich HCP instead of olivine, does this suggest that the entire P58 unit also have similar mineral abundances? We selected some small-sized fresh craters in P58 unit. These craters show stronger absorption bands than the surrounding mature soils, appearing in red hue on the M^3^ IBD map (Fig. 1a). Bright ejecta rays and abundant rocks of these fresh craters can be also observed in the Lunar Reconnaissance Orbiter Camera (LROC) images. Their corresponding orbital M^3^ spectra (dark gray lines in Fig. 6b) were compared with the laboratory-measured spectra of the CE-5 samples. To highlight the discrepancy of the Em basalts from the common Im basalts, some spectra from the fresh craters in the Imbrium-aged older mare basalt regions located to the west of the P58 unit were also extracted (light gray lines in Fig. 6b). On the M^3^ IBD map, the older mare basalts show in green-yellow hue. The root mean squared error (RMSE) of the spectra from P58 unit and nearby Im older basalts with the average spectrum of CE-5 samples are 0.031 and 0.080 (Supplementary Table 3), respectively, indicating the overall spectral shapes of the spectra from P58 unit are closer to those of CE-5 samples. Relative to the nearby Im basalts, weakening of the 2 μm bands is evident in both spectra of CE-5 samples and P58 unit basalts. In addition, the band centers and BAR_1.2μm_ of P58 unit basalts are in accordance with CE-5 samples and much different from nearby Im older basalts (Fig. 3), namely, P58 unit basalts are very likely dominated by similar iron-rich HCP as CE-5 samples rather than olivine.

Although our laboratory spectroscopic measurement was completed on a small amount of CE-5 returned sample (~300 mg), given that CE-5 samples are generally homogeneous^33^ and other study on different CE-5 sub samples have drawn consistent conclusions^31,47,48^, we believe that the ~300 mg sample we analyzed are representative of most CE-5 returned samples. The laboratory measured TiO_2_ (5 wt%) and FeO (22.5 wt%) contents^33^ of the ~300 mg CE-5 samples are also comparable to those of P58 unit estimated from remote sensing data^28^ (Supplementary Fig. 1b, c). Thus, benefiting from spectroscopic measurement, the spectral similarity of the ~300 mg CE-5 samples and P58 unit basalts strongly suggests that the mineralogy and petrology of CE-5 returned samples can be at least generalized to the entire P58 unit.

### Mineralogy and petrogenesis of lunar late-stage basalts

CE-5 returned samples provide a crucial ground truth to our remote observations, which can aid in revising previous hypotheses regarding the mineralogy of the distinct lunar late-stage basalt regions (Supplementary Fig. 1). On the basis of remote spectral interpretation, late-stage basalts were previously recognized as olivine-rich^14,16–18^. Zhang et al.^18^ extracted numerous spectra from every late-stage basalt unit (including P58 unit which was named as U2 in ref. 18) and found their spectral characteristics are very similar. They all have strong, broad, and asymmetric 1 μm absorptions with distinct secondary absorptions around 1.2 μm and weak 2 μm absorptions. The band centers of all these spectra also occur at longer wavelengths (0.986–1.037 μm for 1 μm absorptions, 2.154–2.235 μm for 2 μm absorptions). However, our laboratory analysis on the CE-5 samples prompts us that such spectral characteristics should indicate the likely presence of iron-rich HCP. It is reasonable to suspect that the mineralogy of lunar late-stage basalts is generally dominated by iron-rich HCP rather than olivine, and the variation of the band centers may originate from the variable compositions of HCP containing in different units. Besides, the FeO and TiO_2_ contents of the late-stage basalts are also variable among different units, but are relatively high as a whole compared to their nearby older basalts (Supplementary Fig. 1b, c). The consistent chemical and mineralogical characteristics of lunar late-stage basalts imply that their petrogenesis could be identical. Analysis on the rare-earth elements (REE) patterns and strontium–neodymium (Sr-Nd) isotopes of CE-5 samples may suggest that the CE-5 basalts were formed by low-degree partial melting of the depleted mantle followed by moderate- to high-degree fractional crystallization^47,49^. The possible presence of iron-rich HCP in all lunar late-stage basalts, together with relatively high FeO and TiO_2_ abundance, is also in accordance with the feature of those evolved magmatic products. Accordingly, we speculate that lunar late-stage basalts may have the same petrogenesis as revealed by CE-5 samples. The variation among different late-stage mare units may be attributed to multiple mantle sources or different degrees of the fractional crystallization.

The late-stage basalts are the products of young mare volcanism on the Moon, overlapping a large portion of the nearside mare region (Supplementary Fig. 1a). Their ages were generally dated as younger than 3.0 Ga based on the CSFD method^4,5^. Though the absolute ages of the younger basalts may need to be updated due to the recently dated CE-5 samples^50,51^, their relative ages still reveal that this late-stage mare volcanism could last >1.0 Ga^5,22^. A long-lived source of energy, which kept the melt zones in the lunar mantle from solidifying, could be required to maintain this longevity of mare volcanism over a large area. Furthermore, the lower eruption frequency^4,5,22^ and thinner thickness^6,52,53^ of late-stage basalts compared to the Im old mare basalts (tens of meters vs. several hundreds of meters) suggest that the source energy of late-stage basalts could be considerably weaker relative to that of Im old basalts. Some potential mechanisms that could be involved here to account for such a heat source, including giant impact, enrichment of KREEP (potassium, rare-earth elements and phosphorus)-rich materials, megaregolith and tide. Basin-forming impact can induce pressure release melting of the mantle^54^, but the impact-related heat may not last for such long time. KREEP-rich materials were once considered to account for generating the young volcanism^55–57^. CE-5 basalts truly have relatively high abundance of incompatible trace elements and their pattern is KREEP-like, but their concentrations do not reach the levels of KREEP basalts^31,33,34,47–49^. Nevertheless, the involvement of KREEP materials in the evolution of late-stage basalts based on CE-5 samples remains controversial^31,47,48^. Megaregolith could be one of the reasonable sources^58^, which is an insulating layer coating the lunar surface and keeps the Moon’s interior warm over time. One other plausible heating source is from the liberational tides. Even though tidal variation becomes progressively smaller with decreasing latitudinal migration and increasing Earth-Moon distance during the Eratosthenian period, tidal dissipation may still act at the locations consistent with the occurrence of younger mare basalts^59–61^. Both hypotheses of megaregolith and tide may appear to meet the requirements of the heat source in the extent and duration of late-stage mare volcanism, but further evidence and investigations are still strongly needed and deserved. More samples collected from late-stage mare basalt regions by future sample-return mission will help to finally confirm these hypotheses.

In summary, our comprehensive spectroscopic, XRD, and chemical analyses demonstrate that the special spectral features of CE-5 samples are attributed to the presence of high abundance of iron-rich high-Ca pyroxene rather than olivine, and similar spectral features may also occur in some other evolved mare basalt and soil samples with high titanium contents. Spectral comparison between CE-5 samples and the landing area (P58 unit) as well as other late-stage mare basalt regions suggests their similar mineralogy and petrogenesis. Our work would shed light on the future study of lunar late-stage mare basalts and provide a constraint on the origin and source energy properties of young lunar volcanism.

## Methods

### Preparation of Chang’E-5 lunar soil samples

CE-5 scooped lunar soils were initially packed in a sealed container. After returned to the laboratory, these scooped samples were first transferred into another 16 cm square stainless-steel container (CE5C0000), in which they were thoroughly mixed and large grains (>~1 mm) visible to the naked eye were picked out using tweezers. Then, the samples surface was smoothed and drawn into 16 squares. Ten of these squares were randomly selected, and samples within each square were scooped and placed into ten corresponding sample bottles (CE5C0100 to CE5C1000). After one sampling process, the remaining samples in container CE5C0000 were re-stirred thoroughly, re-smoothed, re-drawn into 16 squares, and re-scooped randomly. This process was repeated several times until all soils in container CE5C0000 were evenly separated into 10 sample bottles (CE5C0100 to CE5C1000). The sampling process ensures a high degree of sample homogeneity among different bottles^33^. All these processes were conducted in a nitrogen-filled glove box.

Three CE-5 scooped soil samples were used in this study. CE5C-S1 is from the CE-5 soil CE5C0800YJFM001, and CE5C-S2 and CE5C-S3 are from the CE-5 soil CE5C0100YJFM002. CE5C0800YJFM001 and CE5C0100YJFM002 are from bottle CE5C0800 and CE5C0100, respectively. During the sample preparation, we first placed each soil sample into the circular groove (2 cm wide, 5 mm deep) of a glassy dish, and then the surface of the samples was lightly smoothed by a piece of quartz glass for subsequent spectroscopic and XRD analysis. The amount of each sample is about 100 mg.

### Spectroscopic measurement of Chang’E-5 soil samples

The spectroscopic measurements of the lunar soil samples were performed in the darkroom. The instrument used was ASD FieldSpec 4. For each sample, one sample dish containing lunar soils was first placed on a platform sprayed with light-absorbing material, which formed a diffused reflection surface with its hemispherical reflectance <0.02. This minimized the effect of scattered light on the measurement results. Then, the viewing geometry of measurement was set to 30° incidence and 0° emergence angle to guarantee that the laboratory measured data have the same viewing geometry as the remote sensing data, which facilitates subsequent spectra comparison. The spectral resolution of ASD is 3 nm@700 nm and 8 nm@1400/2100 nm. The height of the ASD probe was also adjusted to ensure that the soil sample fills the field of view of the ASD probe and that the ASD probe does not cast a shadow on the sample. Finally, 20 reflectance spectra from 450 nm to 2500 nm were obtained for the sample. These processes were conducted for each of the three CE-5 lunar soil samples. In this study, the effect of the orientation of the soil sample on the measured spectra was also evaluated by comparing the measured spectra of sample CE5C-S1 before and after 180° rotation. Our result shows that the spectrum of the rotated sample agrees well with that of sample without rotation (Supplementary Fig. 5). The influence of sample orientation on the spectral shape is not significant.

### X-ray diffraction measurement of Chang’E-5 soil samples

Identification and quantification of the mineral phases of CE-5 soil samples were analyzed by a Bruker D8 Advanced X-ray diffraction instrument and using the whole-pattern Rietveld refinement method. Before conducting XRD analysis, each sample was first stirred thoroughly for at least 1 h to ensure the homogenous mixing and to avoid the preferred orientation of soil grains. Then, the surface of the stirred sample was smoothed with a piece of quartz glass and XRD measurement was performed. For each sample, we repeated this stirring, smoothing, and XRD measurement process 20 times, and the obtained 20 XRD measurements were added up to further eliminate the influence of sample grains’ preferred orientation on the output result of the instrument. The XRD measurement conditions were set as following: 2θ angle ranges from 5° to 90° with the increment set to be 0.015°, the time for each increment is 0.5 s, and the whole-pattern measurement of each sample took ~1 h. A sintered alumina disc (The Standard Reference Material (SRM 1976a)) was used in calibration of X-ray diffraction instrument with respect to diffraction peak position and intensity as a function of 2θ angle.

The JADE software was applied to the measured diffraction pattern of CE-5 samples to realize the mineral phases identification and quantification. This software first identified mineral phases composing CE-5 samples using the method of Hanawalt and Rinn^62^ based on the standard Powder Diffraction Files (PDF) of each mineral phase in the database of International Center for Diffraction Data (ICDD) (https://www.icdd.com). Then, the whole-pattern Rietveld refinement method^63–65^ was adopted by JADE to quantitatively analyze the abundance of each identified mineral phase. Rietveld method fits the entire measured diffraction pattern rather than just the strongest peaks of the sample. The fitting process iteratively compares the sum of weighted, squared differences between the measured and calculated XRD pattern at every 2θ until their best agreement is obtained. The uncertainty in the derived weight fractions and the effects of sample preferred orientation can be minimized using this method. Rietveld fitting requires initial input crystal structure parameters (unit-cell parameters, space group symmetries) set for each mineral phase within the sample. For this study, these information are also from ICDD PDF, and the corresponding PDF numbers used for each mineral phase are the same as that of refs. 33,66.

The measured diffraction patterns of three CE-5 samples are in good agreement with the fitted data (see Supplementary Fig. 3 of ref. 33). The parameters most commonly used to assess the fit is the weighted-profile residual (R_wp_). Typical values of R_wp_ range from a few percent for very good fitting to 20–30%^64^. The fitting R_wp_ of three CE-5 samples are 6.06%, 5.04%, and 5.77%, respectively. The weight fractions of each mineral phase of three samples are shown in Supplementary Table 1.

The XRD measured abundance of CE-5 samples is in terms of weight percentage (wt%). However, the reported mineral abundance of LSCC soil samples is in term of volume percentage (vol%)^44,67^. To obtain the reliable mineral abundance comparison between CE-5 and LSCC soils, the original XRD analysis result in terms of wt% was converted to vol% using the densities (in g/cm^3^) of 2.68 for plagioclase, 3.40 for augite, 3.38 for pigeonite, 3.27 for forsterite, 4.39 for fayalite, 4.72 for ilmenite, 3.19 for apatite, 2.62 for quartz, and 2.40 for glass. These densities data are from https://www.webmineral.com/, which are also the same as that of ref. 31. The converted vol% of each mineral phase is shown in Supplementary Table 2.

### Processing of spectral data

M^3^ data used in this study are from https://pds-imaging.jpl.nasa.gov/volumes/m3.html (see Supplementary Table 4 for M^3^ image IDs used for creating the mosaic of CE-5 landing area). LSCC and mare basalt spectra are available at Reflectance Experiment Laboratory (RELAB) database (now integrated into https://pds-speclib.rsl.wustl.edu/search.aspx). C-TAPE database is at https://www.uwinnipeg.ca/c-tape/sample-database.html. For each CE-5 soil sample, the average of 20 spectra was used as its mean reflectance. The same data processing was applied for all the spectra used in the study, including the spectra of M^3^ data, LSCC samples, RELAB samples, C-TAPE samples and laboratory measured CE-5 samples. The spectra were first smoothed using the Savitzky-Golay method to reduce the influence of noise^68^. Then, the two-straight-lines method^69^ was adopted for the continuum removal of all spectra used in this study. The two-straight lines were tangent to the left and right shoulders of the absorption bands. For 1 μm absorption, the left tangent point was found within 600 to 800 nm, and the right tangent point varied from 1300 to 1800 nm. Iteratively, one point was taken in each of these two ranges until the line joining the two points completely cover the 1 μm absorption band and this straight line was regarded as the tangent line of 1 μm band. The tangent line of 2 μm band can be found in the same way. The left tangent point varied between 1300 and 1800 nm and the right endpoint was set at 2500 nm (the endpoint of the all spectra used in this study is 2500 nm). The continuum-removed spectrum was obtained by dividing the reflectance value of each band by the corresponding tangent line value. All spectra used in this study were treated the same regarding continuum fits for the 2 µm absorption feature.

To characterize the features of the spectra of M^3^ data, LSCC samples, RELAB samples, C-TAPE samples and laboratory measured CE-5 samples, some basic spectral parameters were calculated (Table 1). Band center is often used to identify the lunar pyroxene-bearing materials. Fourth order polynomials were used to fit the continuum removed spectrum around 1 μm and 2 μm absorption regions, and wavelengths corresponding to the minimums of the fitted lines were regarded as the band centers. Integrated band depth (IBD) refers to integration of the band depths over the spectral subset of an absorption feature, and is often used in analyzing M^3^ spectra. The formula used for the calculation of IBD_1μm_ and IBD_2μm_ are as follows^20,70^:

1 $${{{{{{\rm{IBD}}}}}}}_{1\upmu {{{{{\rm{m}}}}}}}=\mathop{\sum }\limits_{N=0}^{26}\{1-R(789+20N)/{R}_{C}(789+20N)\}$$

2 $${{{{{{\rm{IBD}}}}}}}_{2\upmu {{{{{\rm{m}}}}}}}=\mathop{\sum }\limits_{N=0}^{21}\{1-R(1658+40N)/{R}_{C}(1658+40N)\}$$

Here, *R* is reflectance, *R*_*C*_ is the continuum removed reflectance, and *N* is the number of bands used for the calculation of IBD_1μm_ and IBD_2μm_. IBD_1μm_ represents the band depth between 789 nm and 1308 nm relative to a continuum, whereas IBD_2μm_ is the integrated band depth between 1658 nm and 2498 nm relative to a continuum^20^. Integrated band depth ratio (IBDR) is the ratio of IBD_2 μm_ to IBD_1 μm_. In addition, a special spectral parameter was also calculated to characterize the strength of the 1.2 μm band, which we named as 1.2 μm band area ratio (BAR_1.2 μm_) and is defined by:

3 $${{{{{{\rm{BAR}}}}}}}_{1.2\upmu {{{{{\rm{m}}}}}}}=\frac{{{{{{{\rm{Area}}}}}}}_{1.2\upmu {{{{{\rm{m}}}}}}}}{{{{{{{\rm{Area}}}}}}}_{1.2\upmu {{{{{\rm{m}}}}}}}+{{{{{{\rm{Area}}}}}}}_{2\upmu {{{{{\rm{m}}}}}}}}$$

where Area_1.2μm_ and Area_2μm_ are the areas of 1.2 μm and 2 μm bands, respectively (see Supplementary Fig. 6).

We also performed the error evaluation associated with the spectral band parameters. By treating the 20 spectra of each CE-5 sample as replicate analysis, the 1 µm and 2 µm band centers, IBD_1μm_ and IBD_2μm_, IDBR and BAR_1.2μm_ of each individual spectrum were first derived using above mentioned method. Then, the standard deviation (STD) of each parameter was calculated. Results show that the STDs for all the parameters analyzed are small (Table 1), implying agreement among 20 spectra and thus reliable spectroscopic measurement of CE-5 samples.

The formula for the calculation of RMSE between the spectra of the CE-5 samples and M^3^ data is as follows:

4 $${{{{{\rm{RMSE}}}}}}=\sqrt{\frac{{\sum }_{i=1}^{n}\;{(\widehat{{y}_{i}}-{y}_{i})}^{2}}{n}}$$

Here, *y*_*i*_ is the mean reflectance of three CE-5 samples at band *i*, $\widehat{{y}_{i}}$ is the reflectance of late-stage mare basalts or older mare basalts at band *i*, and *n* is the number of bands. The smaller value of RMSE suggests similar spectral features between the CE-5 samples and fresh craters.

## Supplementary information

Supplementary Information

## Data Availability

Laboratory measured reflectance data for CE5C-S1, CE5C-S2, and CE5C-S3 samples are provided in Source Data files. In addition, the spectral parameters data for pure pyroxene, LSCC soil samples, Apollo mare basalts samples, and M^3^ spectra data derived in this study are also provided in Source Data files. Source data are provided with this paper.

## Source data

Source Data
