# Supplementary material for: Spectral interpretation of late-stage mare basalt mineralogy unveiled by Chang’E-5 samples
Source: Nat Commun. 2022 Oct 10;13:5965. doi: 10.1038/s41467-022-33670-6 (PMC9550791; doi:10.1038/s41467-022-33670-6)
Supplement: Supplementary file 1 — Supplementary Information [file 41467_2022_33670_MOESM1_ESM.pdf]

Supplementary Information

Spectral Interpretation of Late-Stage Mare Basalt Mineralogy Unveiled by Chang'E-5 Samples

Dawei Liu[1#], Xing Wang[1,2#], Jianjun Liu[1,2*], Bin Liu[1*], Xin Ren[1], Yuan Chen[1], Zhaopeng Chen[1,2], Hongbo Zhang[1], Guangliang Zhang[1], Qin Zhou[1], Zhoubin Zhang[1], Qiang Fu[1] & Chunlai Li[1,2*]

1 Key Laboratory of Lunar and Deep Space Exploration, National Astronomical Observatories, Chinese Academy of Sciences, Beijing 100101, China.
2 School of Astronomy and Space Science, University of Chinese Academy of Sciences, Beijing 100049, China.
# These authors contributed equally: Dawei Liu, Xing Wang.
* Corresponding author: Jianjun Liu(liujj@nao.cas.cn); Bin Liu (liub@nao.cas.cn); Chunlai Li (licl@nao.cas.cn).

Supplementary Note 1

Selection of pure pyroxene samples

The samples of pure pyroxene employed in this study can be divided into three groups, including synthetic pyroxenes, terrestrial natural pyroxenes and lunar pyroxenes. All the spectra of these samples are available at Reflectance Experiment Laboratory (RELAB) database (now integrated into https://pds-speclib.rsl.wustl.edu/search.aspx) and the C-TAPE database (https://www.uwinnipeg.ca/c-tape/sample-database.html). The group of synthetic pyroxenes is from Klima et al.[1-3]. Note that synthetic Ca-free orthopyroxenes are excluded here because they have an apparent 1.2 μm absorption which is not commonly seen in natural orthopyroxene. The rapid cooling of synthetic orthopyroxene results in a higher degree of disorder, making more Fe^{2+} occupy the M1 site, whereas Fe^{2+} preferentially occupies the M2 site in natural orthopyroxene. The group of terrestrial natural pyroxenes have been analyzed in many previous studies[4,5]. Natural clinopyroxenes (CPXs) used here are all type-B CPXs considering type-A CPXs have not been found in lunar observations[6]. Detailed descriptions of these natural pyroxenes can be seen at the C-TAPE database. The group of lunar pyroxenes is from the Lunar Rock and Mineral Characterization Consortium (LRMCC)[7]. The lunar pyroxenes are separated from two low-Ti basalts (15058 and 15555) and two high-Ti basalts (70017 and 70035). For all these samples, if measurements of multiple grain sizes are available, we calculated the spectral parameters from each grain size in order to show the possible influence of the grain size on the spectral parameters. The compositions of all pyroxenes used in this study are shown in Supplementary Fig. 7. Note that Ca-saturated pyroxenes are also excluded in this study, because lunar pyroxenes seldom exhibit compositions with Ca >47%[8].

Supplementary Note 2

Variation of the spectral parameters of the mineral mixtures

Here we define olivine, low-Ca clinopyroxene, high-Ca clinopyroxene and pyroxene as OL, LCP, HCP, and PYX, respectively. To test the variation of the spectral parameters when pyroxene is mixed with olivine, we selected four groups of mixture spectra, including LCP+HCP, LCP+HCP+OL, LCP+OL and HCP+OL. The LCP+HCP mixture refers to the mixture of PX-RGM-017 and PX-RGM-018 (Relab sample IDs) and the corresponding spectra can be found in Relab database. The LCP+HCP+OL mixture is the mixture of AG-TJM-008, AG-TJM-009 and AG-TJM-010, and the spectra are in Relab database. The LCP+OL mixtures refer to the mixture of PYX042+OLV003 and mixture of PYX003+OLV022, and their spectra can be found in C-TAPE database. The HCP+OL mixtures consist of the mixtures of PYX036+OLV022, PYX016+OLV003 and PYX 040+OLV020, and are also available in C-TAPE database. The variation of the spectral parameters of these mixtures with the mixing proportions is displayed in Supplementary Fig. 8.

It can be seen that although the relationship between 1 μm and 2 μm band centers is a valid tool to access the bulk pyroxene composition, it is hard to determine whether a mixture spectrum suggests the enrichment of olivine if based only on the relationship of band centers. In many cases, even if the pyroxene/olivine (PYX/OL) ratio is close to 50/50, the band centers of these mixtures do not deviate significantly from the relationship of the band centers established by pure pyroxenes with diverse compositions, especially for the HCP+OL mixtures. Note that the mixing of LCP and HCP follows this relationship of band centers. Considering the absence of 2 μm absorption band in the pure olivine spectrum, for the PYX+OL mixed spectrum, the 2 μm band center from pyroxene is not supposed to be shifted significantly with the increasing of the olivine abundance, and thus the position of 2 μm band center can be regarded as a rough indicator of the pyroxene composition. Supplementary Fig. 8b shows the $BAR_{1.2μm}$ as the function of the position of the 2 μm band center. The white-gray-black points exhibit how the $BAR_{1.2μm}$ of pure pyroxenes varies with their composition. It is important to highlight that the values of $BAR_{1.2μm}$ of all pyroxenes are less than 0.5 except for the Ca-saturated pyroxenes that have been excluded in this work[2]. At the LCP end with shorter 2 μm band centers, the values of $BAR_{1.2μm}$ are close to 0. This is due to the fact that Fe^{2+} cations prefer the M2 site to the M1 site in the well-ordered natural LCP. When the calcium content is low, the M2 site is sufficient to accommodate most Fe^{2+} cations and less Fe^{2+} cations occupy the M1 site. This results in an insignificant 1.2 μm absorption band with a relatively strong 2 μm absorption band, and thus the $BAR_{1.2μm}$ of LCP is small. While at the HCP end with longer 2 μm band centers, the values of $BAR_{1.2μm}$ become larger and more scattered. As the calcium content increases, the composition and structure of pyroxenes become complex. More substitutions of Ca^{2+} cations force more Fe^{2+} cations to be partitioned into M1 site in HCP, leading to the greater $BAR_{1.2μm}$ values. Besides, Fe-rich HCPs generally have greater $BAR_{1.2μm}$ values than Mg-rich HCPs, also because more Fe^{2+} cations could fill the M1 site. In Supplementary Fig. 8b, a series of colored points connected with dashed lines represent a variety of mixtures with different proportions. Note that the 2 μm band centers of the LCP+HCP mixture vary significantly with mixing proportions, but still follow the relationship established by the pure pyroxenes. For the mixture of pyroxene and olivine, the values of $BAR_{1.2μm}$ rise considerably with increasing proportion of olivine, while the 2 μm band centers do not shift as much. In particular, at the HCP end, if the olivine abundance in the mixture is >50%, the value of $BAR_{1.2μm}$ could be greater than 0.5, which substantially exceeds the range of pyroxene.

It should be noted that factors such as data quality may have a small impact on this preliminary method, but will not affect the integrated evaluation. At least for the CE-5 samples with HCP composition and $BAR_{1.2μm}$ value less than 0.5, it indicates that the olivine is not significant, unlike previous claims that the OL/PYX ratio is greater than 1.

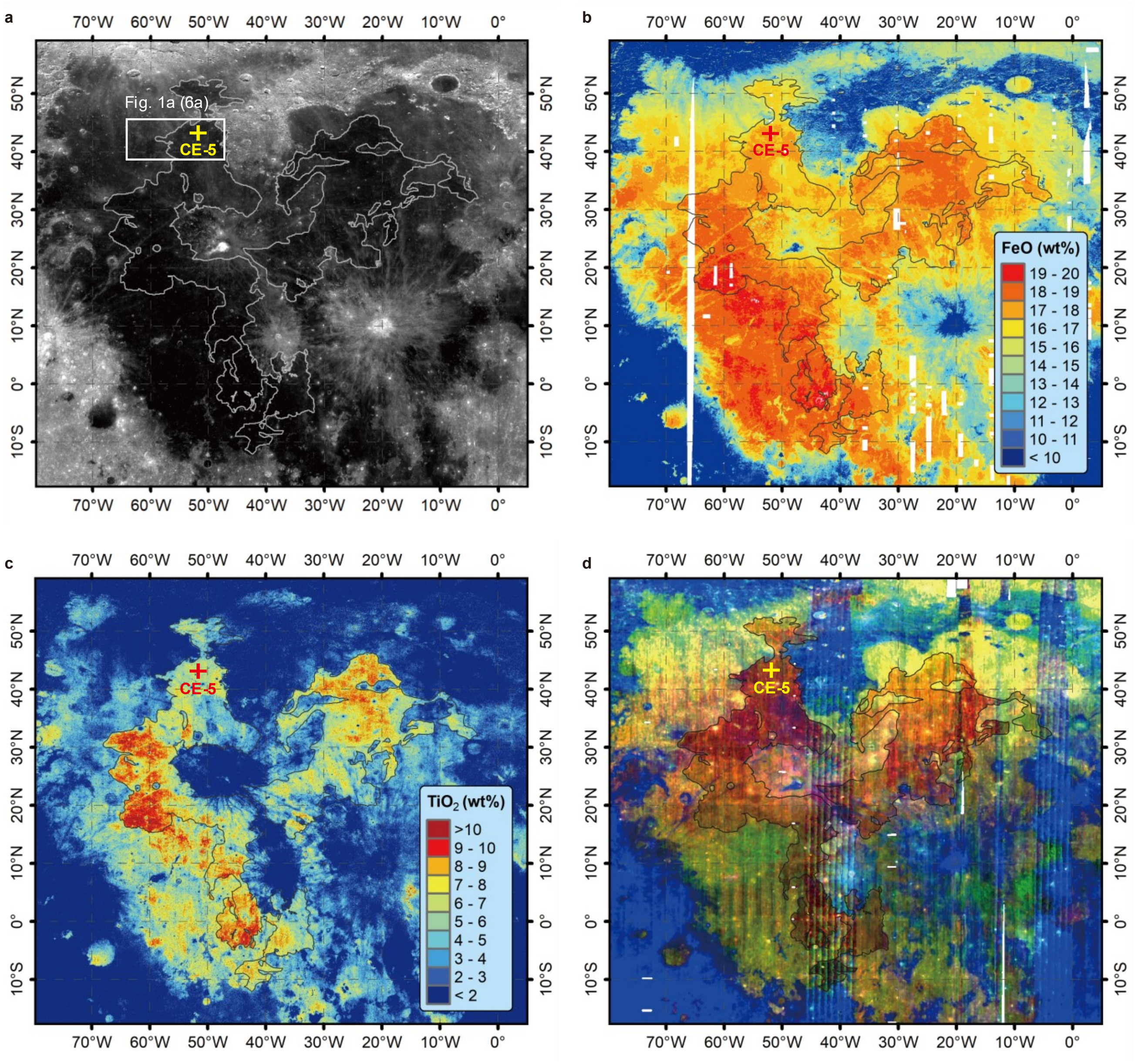

Supplementary Figure 1. Geological backgrounds of lunar late-stage basalts. **a** Chang'E-2 (CE-2) global digital orthophoto model (DOM) (50m resolution) of the lunar late-stage basalts. Lunar late-stage basalts are the dark volcanic flows that is roughly outlined by the gray boundary in the image. The boundary is modified based on Hiesinger et al.[9] and Zhang et al.[10]. The white box indicates the range of Fig.1a and 6a. **b** FeO content of lunar late-stage basalts derived from Clementine multispectral data using method of Lucey et al.[11]. **c** TiO_2 content of lunar late-stage basalts. **d** Moon Mineralogy Mapper (M^3) IBD map of lunar late-stage basalts. The map is mainly displayed by M^3 data collected in OP2C optical period, and the regions which are not covered by OP2C data are supplemented by OP1B data. CE-2 global DOM with 50m resolution data are available at http://www.dx.doi.org/10.12350/CLPDS.GRAS.CE2.DOM-50m.vA. Image IDs used to generate the mosaic of **a** are provided in Supplementary Table 5. Clementine multispectral data can be downloaded from https://astrogeology.usgs.gov/search/map/Moon/Clementine/UVVIS/Lunar_Clementine_UVVIS_WarpMosaic_5Bands_200m. TiO_2 abundance data are available at https://wms.lroc.asu.edu/lroc/view_rdr/WAC_TIO2. M^3 data are available at https://pds-imaging.jpl.nasa.gov/volumes/m3.html.

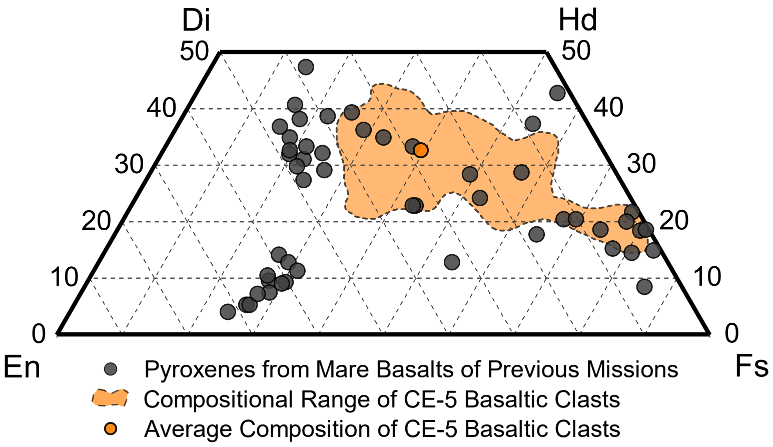

Supplementary Figure 2. Pyroxene quadrilateral showing comparison of pyroxene compositions between CE-5 basaltic clasts and the basalts from previous missions. Pyroxene compositions of CE-5 basaltic clasts are from Li et al.[12]. Pyroxene compositions of mare basalts collected by previous missions can be found in Table 5.2 of Papike et al.[13]. Di, Hd, En, Fs represent diopside, hedenbergite, enstatite and ferrosilite, respectively.

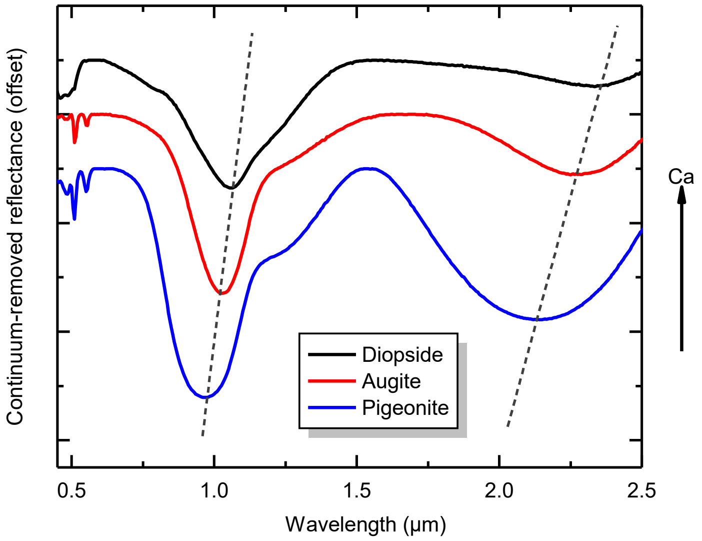

Supplementary Figure 3. Weakening of 2 μm bands with the increasing of Ca content in pyroxenes. The spectra data are from Relab database. The sample IDs for diopside, augite, pigeonite are DL-CMP-037, DL-CMP-085 and DL-CMP-053, respectively. Black arrow shows the direction of increasing Ca content of pyroxenes. Dotted lines indicate the band centers of pyroxenes.

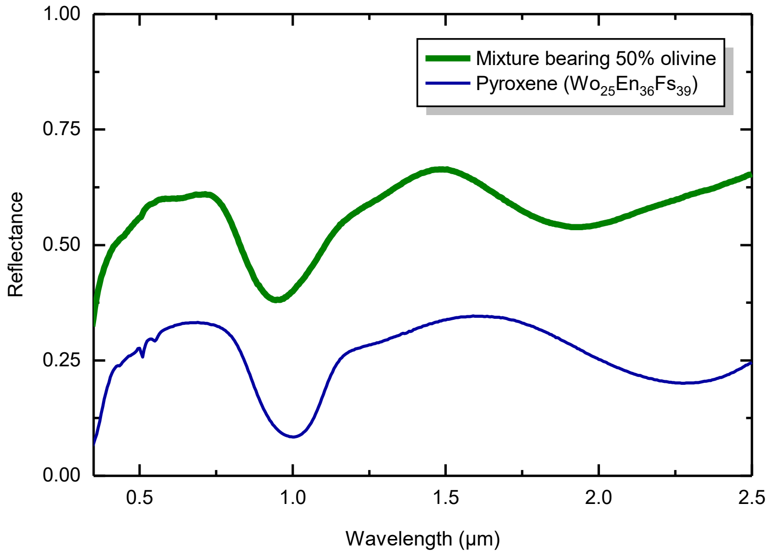

Supplementary Figure 4. Examples of an olivine-bearing mixture spectrum and a pure pyroxene spectrum. It can be seen that the olivine-bearing spectrum is somewhat similar to a pure pyroxene spectrum, especially at the 1.2 μm band. The olivine-bearing mixture spectrum is AG-TJM-038 from Relab database, which is composed of 50% olivine (OL), 25% low-Ca clinopyroxene (LCP) and 25% high-Ca clinopyroxene (HCP). The pure pyroxene spectrum is DL-CMP-057-A from Relab database with composition of $Wo_{25}En_{36}Fs_{39}$.

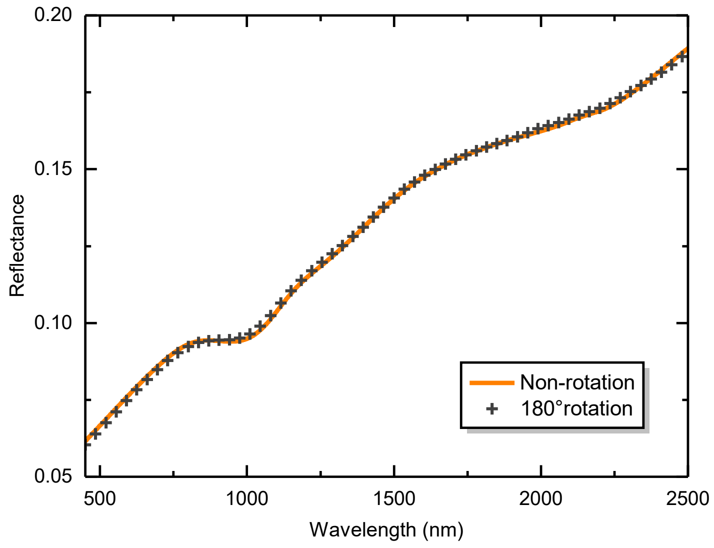

Supplementary Figure 5. Comparison of the CE5C-S1 sample spectra with non-rotation and with 180° rotation. The spectrum of the rotated sample agrees well with that of sample without rotation, suggesting the influence of sample orientation on the spectral shape is not significant.

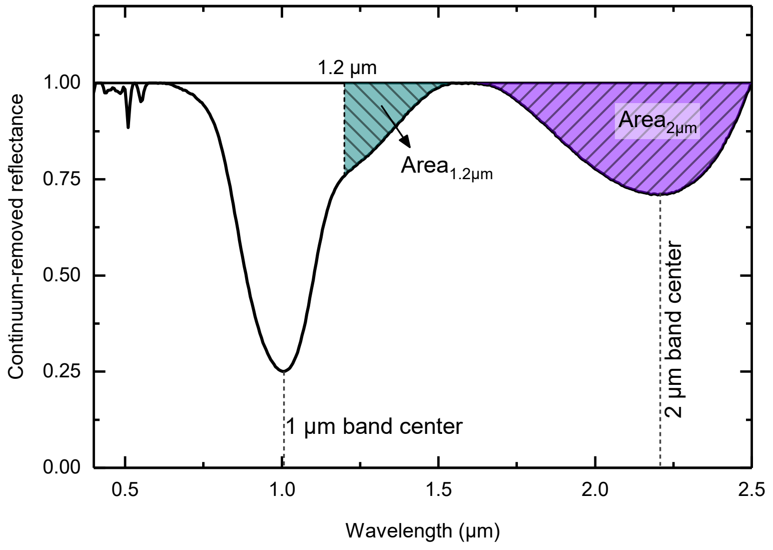

Supplementary Figure 6. Diagram for calculating spectral parameters based on a continuum removed spectrum. 1 µm and 2 µm band centers are the wavelength positions of the minimums of fourth order polynomial fits to the 1 µm and 2 µm absorption bands after continuum removal. $Area_{1.2\mu m}$ (cyan colored region) indicates the geometric area enclosed by 1.2 µm absorption and reflectance equal to 1, and $Area_{2\mu m}$ (purple colored region) refers to the geometric area enclosed by 2 µm absorption and reflectance equal to 1.

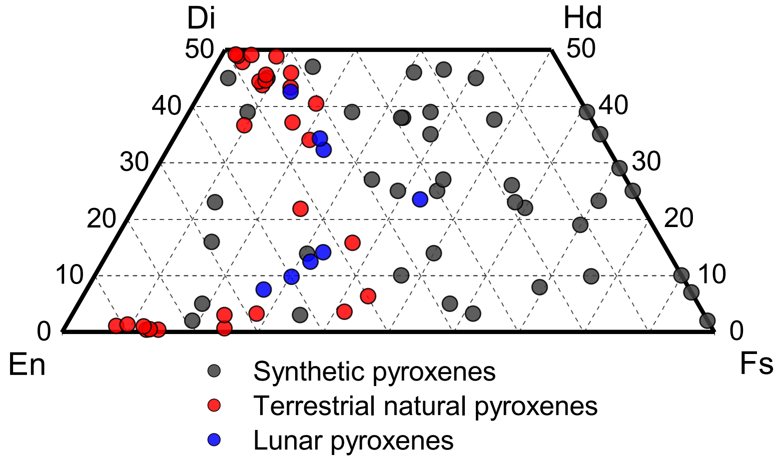

Supplementary Figure 7. Pyroxene compositions used in this study with respect to pyroxene quadrilateral. Different colored solid circles represent different groups of pyroxenes. Di, Hd, En, Fs represent diopside, hedenbergite, enstatite and ferrosilite, respectively.

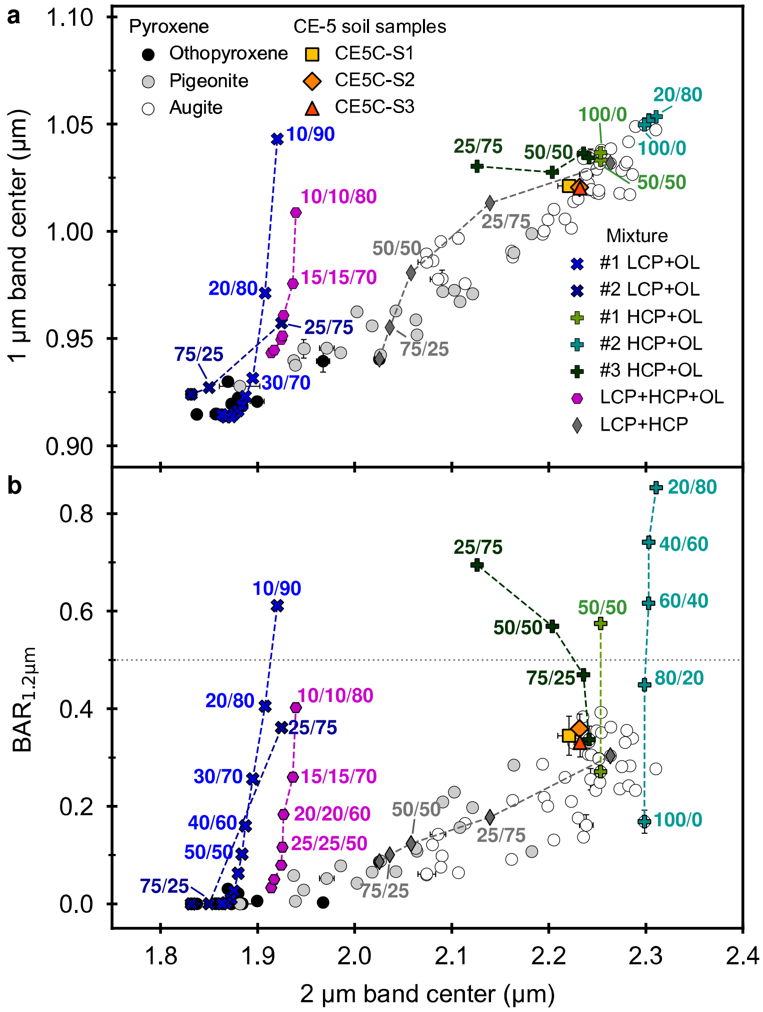

Supplementary Figure 8. Variations of the spectral parameters of the olivine-bearing mixtures. a 1 µm and 2 µm band centers. **b** 1.2 µm band area ratio ($BAR_{1.2\mu m}$) and 2 µm band center. The white-gray-black points are from pure pyroxenes that are described in Supplementary Note 1 and are the same as those used in Fig. 3. Some pure pyroxene points have error bars that indicate the standard deviation of the samples with different grain sizes, and the points without error bars are derived from a single measurement. The error bars of CE-5 soil samples are the standard deviation of the derived spectral parameters. LCP, HCP, OL represent low-Ca clinopyroxene, high-Ca clinopyroxene and olivine, respectively. A series of colored points connected with dashed lines represent a variety of olivine-bearing mixtures with different proportions. #1 LCP+OL, #2 LCP+OL, #1 HCP+OL, #2 HCP+OL and #3 HCP+OL refer to PYX042+OLV003, PYX003+OLV022, PYX036+OLV022, PYX016+OLV003 and PYX040+OLV020 in C-TAPE database, respectively. LCP+HCP and LCP+HCP+OL are the mixture of PX-RGM-017 (LCP) and PX-RGM-018 (HCP) and the mixture of AG-TJM-008 (OL), AG-TJM-009 (LCP) and AG-TJM-010 (HCP) in Relab database, respectively. The values marked on the figure are the modal percentages of LCP/OL for the LCP+OL mixtures, HCP/OL for the HCP+OL mixtures, LCP/HCP/OL for the LCP+HCP+OL mixture and LCP/HCP for the LCP+HCP mixture, respectively. The horizontal dotted line in **b** represents $BAR_{1.2\mu m}$=0.5.

Supplementary Table 1. Mineral phases and their weight percentage of CE-5 soil samples (wt %).

Phase	Samples wt% (σ)			
	CE5C-S1	CE5C-S2	CE5C-S3	Mean
Plagioclase	29.1 (0.6)	31.8 (0.4)	29.3 (0.5)	30.1
Augite	28.8 (0.6)	35.0 (0.7)	28.8 (0.6)	30.9
Pigeonite	13.3 (0.5)	9.1 (0.4)	10.9 (0.4)	11.1
Forsterite	2.0 (0.2)	1.5 (0.1)	1.4 (0.1)	1.6
Fayalite	3.5 (0.2)	4.9 (0.2)	4.0 (0.2)	4.1
Ilmenite	4.6 (0.1)	4.3 (0.1)	4.6 (0.1)	4.5
Apatite	0.1 (0.0)	1.4 (0.1)	0.7 (0.1)	0.7
Quartz	0.4 (0.1)	0.4 (0.1)	0.3 (0.1)	0.4
Glass	18.2 (1.2)	11.6 (1.0)	20.0 (1.3)	16.6

Note：Glass here represents the sum of agglutinitic glass and volcanic glass. Data are the same as Li et al.[12].

Supplementary Table 2. Converted volume percentage of mineral phases in CE-5 soil samples (vol %).

Phase	Samples vol%			
	CE5C-S1	CE5C-S2	CE5C-S3	Mean
Plagioclase	32.5	36.2	32.5	33.7
Augite	25.4	31.4	25.2	27.3
Pigeonite	11.8	8.2	9.6	9.9
Forsterite	1.8	1.4	1.3	1.5
Fayalite	2.4	3.4	2.7	2.8
Ilmenite	2.9	2.8	2.9	2.9
Apatite	0.1	1.3	0.7	0.7
Quartz	0.5	0.5	0.3	0.4
Glass	22.7	14.8	24.8	20.7

Supplementary Table 3. Locations of the fresh craters for extracting M^3 spectra, and their corresponding RMSEs with respect to the mean spectra of CE-5 samples.

Age	ID	Longitude	Latitude	RMSE	Age	ID	Longitude	Latitude	RMSE
Em	Em1	-51.654	44.451	0.025	Im	Im1	-64.490	40.831	0.059
	Em2	-48.823	43.590	0.035		Im2	-60.642	45.158	0.087
	Em3	-52.858	44.259	0.030		Im3	-65.141	43.743	0.090
	Em4	-53.513	42.983	0.030		Im4	-63.090	42.648	0.075
	Em5	-51.529	41.754	0.043		Im5	-59.600	43.222	0.075
	Em6	-49.325	42.935	0.042		Im6	-60.575	45.077	0.078
	Em7	-48.594	42.839	0.032		Im7	-62.516	45.306	0.0518
	Em8	-48.713	40.650	0.033		Im8	-58.333	44.341	0.046
	Em9	-49.942	40.937	0.029		Im9	-55.435	45.354	0.119
	Em10	-50.492	40.090	0.029		Im10	-60.532	40.774	0.085
	Em11	-53.403	41.056	0.034		Im11	-58.658	43.863	0.119
	Em12	-52.304	40.267	0.033		Im12	-59.518	43.002	0.074
	Em13	-51.697	41.290	0.030		Im13	-55.689	45.024	0.083
Mean				0.031	Mean				0.080

Supplementary Table 4. IDs of M^3 images used for generating the mosaic of CE-5 landing area.

No.	M^3 image IDs
1	M3G20090612T183813_V01_RFL.IMG
2	M3G20090613T120320_V01_RFL.IMG
3	M3G20090613T073612_V01_RFL.IMG
4	M3G20090613T032520_V01_RFL.IMG
5	M3G20090612T101600_V01_RFL.IMG
6	M3G20090612T143522_V01_RFL.IMG
7	M3G20090612T230542_V01_RFL.IMG

Supplementary Table 5. Image IDs of CE-2 DOM with 50m resolution used to generate the mosaic of lunar late-stage mare basalts.

No.	Image IDs	No.	Image IDs
1	CE2_GRAS_DOM_50m_C003_63N105W_A.tif	23	CE2_GRAS_DOM_50m_F011_21N009E_A.tif
2	CE2_GRAS_DOM_50m_C004_63N075W_A.tif	24	CE2_GRAS_DOM_50m_G005_07N099W_A.tif
3	CE2_GRAS_DOM_50m_C005_63N045W_A.tif	25	CE2_GRAS_DOM_50m_G006_07N081W_A.tif
4	CE2_GRAS_DOM_50m_C006_63N015W_A.tif	26	CE2_GRAS_DOM_50m_G007_07N063W_A.tif
5	CE2_GRAS_DOM_50m_C007_63N015E_A.tif	27	CE2_GRAS_DOM_50m_G008_07N045W_A.tif
6	CE2_GRAS_DOM_50m_D004_49N096W_A.tif	28	CE2_GRAS_DOM_50m_G009_07N027W_A.tif
7	CE2_GRAS_DOM_50m_D005_49N072W_A.tif	29	CE2_GRAS_DOM_50m_G010_07N009W_A.tif
8	CE2_GRAS_DOM_50m_D006_49N048W_A.tif	30	CE2_GRAS_DOM_50m_G011_07N009E_A.tif
9	CE2_GRAS_DOM_50m_D007_49N024W_A.tif	31	CE2_GRAS_DOM_50m_H005_07S099W_A.tif
10	CE2_GRAS_DOM_50m_D008_49N000W_A.tif	32	CE2_GRAS_DOM_50m_H006_07S081W_A.tif
11	CE2_GRAS_DOM_50m_E005_35N090W_A.tif	33	CE2_GRAS_DOM_50m_H007_07S063W_A.tif
12	CE2_GRAS_DOM_50m_E006_35N070W_A.tif	34	CE2_GRAS_DOM_50m_H008_07S045W_A.tif
13	CE2_GRAS_DOM_50m_E007_35N050W_A.tif	35	CE2_GRAS_DOM_50m_H009_07S027W_A.tif
14	CE2_GRAS_DOM_50m_E008_35N030W_A.tif	36	CE2_GRAS_DOM_50m_H010_07S009W_A.tif
15	CE2_GRAS_DOM_50m_E009_35N010W_A.tif	37	CE2_GRAS_DOM_50m_H011_07S009E_A.tif
16	CE2_GRAS_DOM_50m_E010_35N010E_A.tif	38	CE2_GRAS_DOM_50m_I005_21S099W_A.tif
17	CE2_GRAS_DOM_50m_F005_21N099W_A.tif	39	CE2_GRAS_DOM_50m_I006_21S081W_A.tif
18	CE2_GRAS_DOM_50m_F006_21N081W_A.tif	40	CE2_GRAS_DOM_50m_I007_21S063W_A.tif
19	CE2_GRAS_DOM_50m_F007_21N063W_A.tif	41	CE2_GRAS_DOM_50m_I008_21S045W_A.tif
20	CE2_GRAS_DOM_50m_F008_21N045W_A.tif	42	CE2_GRAS_DOM_50m_I009_21S027W_A.tif
21	CE2_GRAS_DOM_50m_F009_21N027W_A.tif	43	CE2_GRAS_DOM_50m_I010_21S009W_A.tif
22	CE2_GRAS_DOM_50m_F010_21N009W_A.tif	44	CE2_GRAS_DOM_50m_I011_21S009E_A.tif

Supplementary References

1. Klima, R. L., Pieters, C. M. & Dyar, M. D. Spectroscopy of synthetic Mg-Fe pyroxenes I: Spin-allowed and spin-forbidden crystal field bands in the visible and near-infrared. *Meteorit. Planet. Sci.* **42**, 235-253 (2007).
2. Klima, R. L., Pieters, C. M. & Dyar, M. D. Characterization of the 1.2 μm M1 pyroxene band: Extracting cooling history from near-IR spectra of pyroxenes and pyroxene-dominated rocks. *Meteorit. Planet. Sci.* **43**, 1591-1604 (2008).
3. Klima, R. L., Dyar, M. D. & Pieters, C. M. Near-infrared spectra of clinopyroxenes: Effects of calcium content and crystal structure. *Meteorit. Planet. Sci.* **46**, 379-395 (2011).
4. Adams, J. B. Visible and near-infrared diffuse reflectance spectra of pyroxenes as applied to remote sensing of solid objects in the solar system. *J. Geophys. Res.* **79**, 4829-4836 (1974).
5. Cloutis, E. A. & Gaffey, M. J. Pyroxene spectroscopy revisited: Spectral-compositional correlations and relationship to geothermometry. *J. Geophys. Res.-Planets* **96**, 22809-22826 (1991).
6. Zhang, X., & Cloutis, E. Near-infrared Spectra of Lunar Ferrous Mineral Mixtures. *Earth Space Sci.* **8**, e2020EA001153 (2021).
7. Isaacson, P. J. et al. The lunar rock and mineral characterization consortium: Deconstruction and integrated mineralogical, petrologic, and spectroscopic analyses of mare basalts. *Meteorit. Planet. Sci.* **46**, 228-251 (2011).
8. Moriarty III, D. P. & Pieters, C. M. Complexities in pyroxene compositions derived from absorption band centers: Examples from Apollo samples, HED meteorites, synthetic pure pyroxenes, and remote sensing data. *Meteorit. Planet. Sci.* **51**, 207-234 (2016).
9. Hiesinger, H., Head III, J. W., Wolf, U., Jaumann, R. & Neukum, G. Ages and stratigraphy of mare basalts in Oceanus Procellarum, Mare Nubium, Mare Cognitum, and Mare Insularum. *J. Geophys. Res.-Planets* **108,** E7 (2003).
10. Zhang, X. et al. Mineralogical variation of the late stage mare basalts. *J. Geophys. Res.-Planets* **121**, 2063-2080 (2016).
11. Lucey, P. G., Taylor, G. J. & Malaret, E. Abundance and distribution of iron on the Moon. *Science* **268,** 1150-1153 (1995).
12. Li, C. et al. Characteristics of the lunar samples returned by Chang'E-5 mission. *Nat. Sci. Rev.* **9,** nwab188 (2022).
13. Papike, J., Taylor, L. & Simon, S. Lunar minerals. *Lunar Sourcebook: A User's Guide to the Moon Ch. 5* (Cambridge University Press, 1991).
